# Cyclin-Dependent Kinases 4/6 Inhibitors in Breast Cancer: Current Status, Resistance, and Combination Strategies

**DOI:** 10.7150/jca.32628

**Published:** 2019-08-29

**Authors:** Ying Niu, Junnan Xu, Tao Sun

**Affiliations:** 1 Department of Medical Oncology, Cancer Hospital of China Medical University, Liaoning Cancer Hospital and Institute, No. 44 Xiaoheyan Road, Dadong, Shenyang, Liaoning 110042, P.R. China; 2 Key Laboratory of Liaoning Breast Cancer Research, Shenyang, Liaoning 110042, P.R. China

**Keywords:** CDK 4/6 inhibitors, breast cancer, clinical trials, drug resistance, combination treatment

## Abstract

Dysregulated activation of the cyclin-dependent kinases (CDKs) 4/6, leading to uncontrolled cell division, is hallmark of cancers. Further study of the cell cycle will advance the cancer treatment. As powerful and effective drugs, inhibitors of CDK 4/6 have been widely used in clinical practice for several malignancies, particularly against breast cancers driven by the estrogen receptor (ER). Three CDK4/6 inhibitors, including palbociclib (PD0332991), ribociclib (LEE011) and abemaciclib (LY2835219), have been approved by the US Food and Drug Administration (FDA) for the treatment of hormone receptor-positive, human epidermal growth factor receptor 2-negative advanced or metastatic breast cancer. However, CDK4/6 inhibitors act downstream of many mitogenic signaling pathways, and this has implications for resistance. It is worth to note that the mechanisms of resistance are not very clear. Up to now, a small number of preclinical and clinical studies have explored potential mechanisms of CDK4/6 inhibitors resistance in breast cancer. On this basis, rational and effective combination therapy is under development. Here we review the current knowledge about the mechanisms and efficacy of CDK4/6 inhibitors, and summarize data on resistance mechanisms to make future combination therapies more accurate and reasonable.

## 1. Introduction

**Figure 1:**
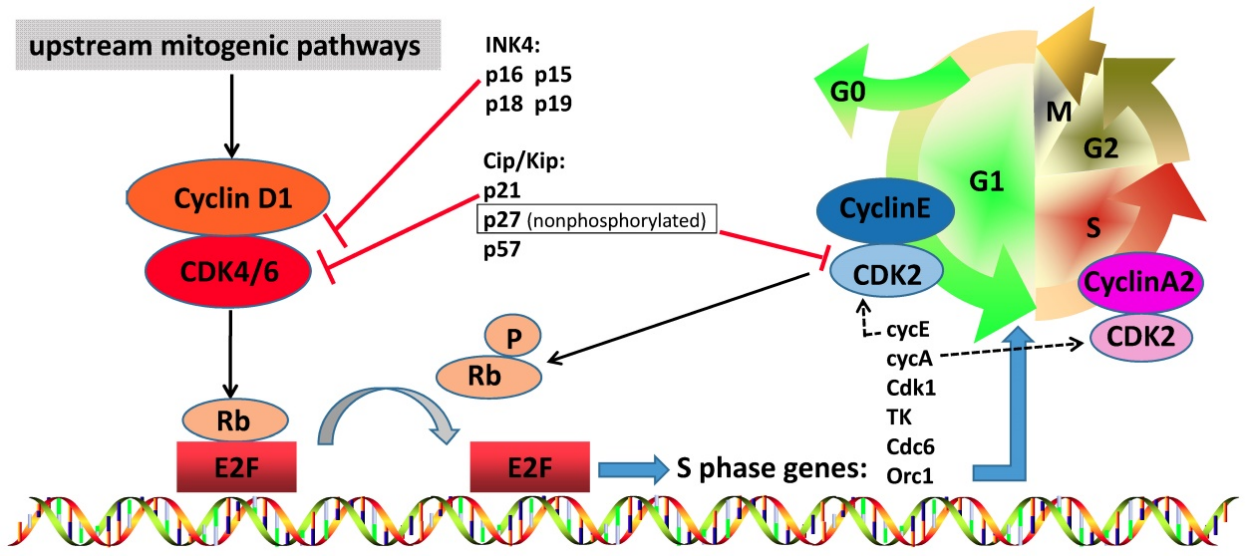
** The role of cyclin D-CDK4/6-INK4-Rb pathway in breast cancer.** CDK4 and CDK6 play a very important role in cell cycle entry, including cross talk with other oncogenic signal pathways. When the cell prepares to initiate DNA synthesis, upstream mitogenic pathways increase cyclin D1 levels, which may activate CDK4/6. Active complex of CDK4/6 and cyclin D1 phosphorylates and inactivates RB protein, which is then phosphorylated by other complexes such as cyclin E-CDK2 in the late G1 phase. Phosphorylated RB releases transcription factor E2F, permitting the up-regulation of E2F activation and transcription of client genes required for cell cycle G1/S transition. Cyclin A2-CDK2 complex increases and phosphorylates proteins involved in DNA synthesis, thereby driving S phase progression. The kinase activity of CDK4/6 is tightly suppressed by endogenous inhibitors, such as Cip/Kip family members (p21Cip1, nonphosphorylated p27Kip1 and p57Kip2) and INK4 family proteins (p16INK4a, p15INK4b, p18INK4c and p19INK4d), and pharmacologic CDK4/6 inhibitors. And nonphosphorylated p27 suppress the CDK2 and has an oncogenic function to maintain cyclin D-CDK4 activity.

**Figure 2:**
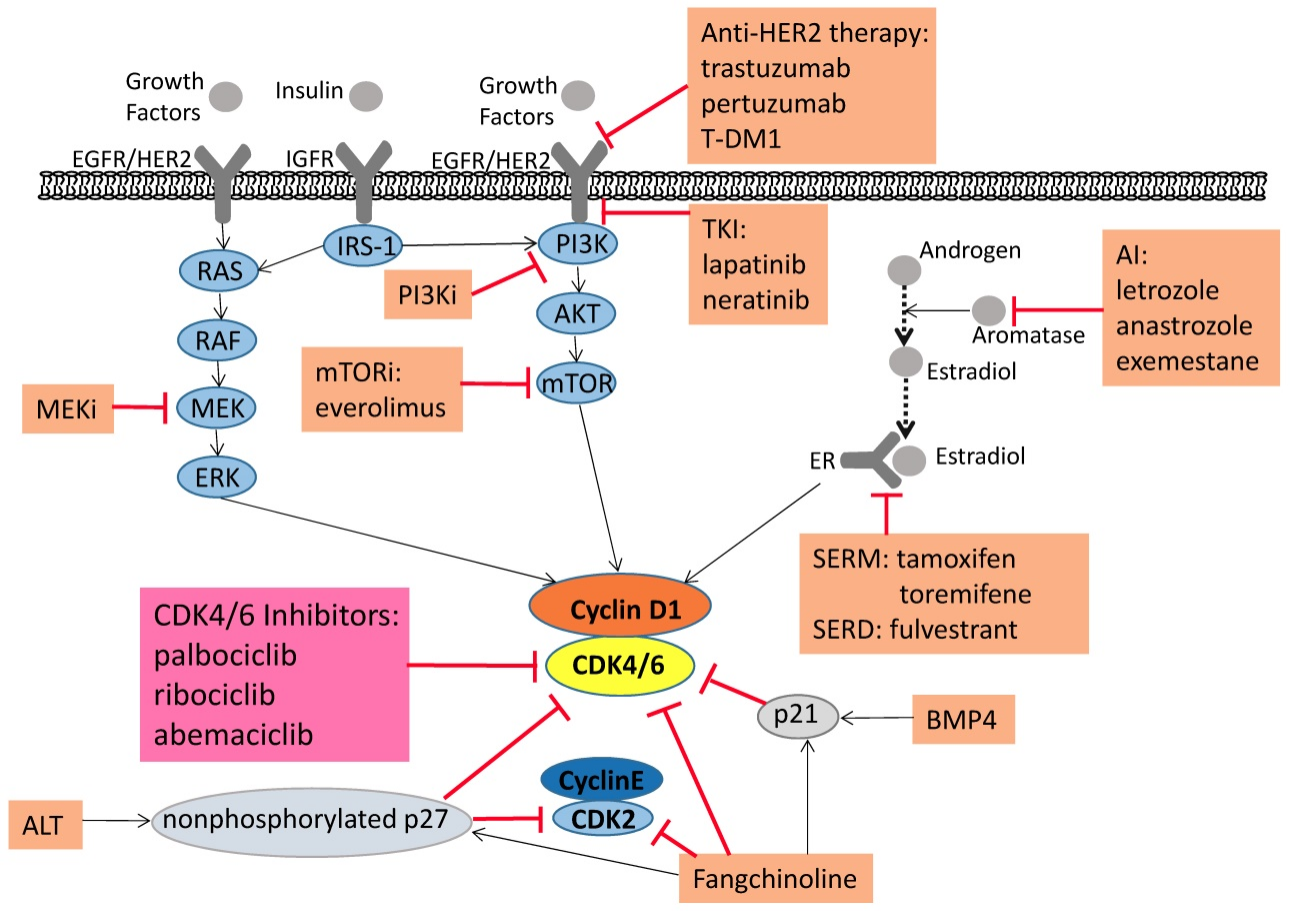
** Signaling pathways associated with tumorigenesis and combined treatments that alleviate drug resistance.** Pharmaceutical CDK4/6 inhibitors palbociclib, ribociclib, and abemaciclib directly inhibit CDK4/6 activity. Moreover, the upstream mitogenic forces, including the canonical RAS-RAF-MEK-ERK pathway, heightened activity of the HER2-PI3K-AKT-mTOR axis, increase the cyclin D1 levels, activating CDK4/6 and promoting cellular progression to the S phase. Because of this foundation, PI3K, mTOR and MEK inhibitors induce synergistic anti-proliferative and pro-apoptotic effects, which lead to more durable cell cycle arrest and a delay to the onset of resistance. The Aromatase Inhibitors (AI), which inhibit the transformation of androgen into estradiol, thereby suppress breast cancer cell growth. Selective estrogen receptor modulator (SERM) and selective estrogen receptor downregulator (SERD) can affect estrogen receptors to produce the same inhibitory effect on tumor cells. ALT can keep p27 in a non-phosphorylated state, which is a stable form, and reduce both CDK2 and CDK4 activity. BMP4 and Fangchinoline can upregulate p21. Fangchinoline not only increases the level of CKIs (p21 and p27), but also inhibits cyclin D1/D3/E and CDK2/4/6. The ALT, BMP4 and Fangchinoline are still under preclinical study. In addition, clinical studies on the combination of CDK4/6 inhibitors with anti-HER2 therapy and immunotherapy are under way.

The cell cycle is a critical regulator of cell proliferation, growth and division after DNA damage. It controls the transition from the quiescent state (G0 phase) to cell proliferation and passes through checkpoints 1. To enter DNA synthesis (S phase), all cells must activate cyclin-dependent kinases (CDKs), which require binding to a cyclin subunit to become catalytic active. The D-type cyclins and their partner kinases, CDK 4 and 6, play important role in cell cycle (Figure 1) 2, 3. Activation of upstream mitogenic pathways, including PI3K-AKT-mTOR, RAS-RAF-MEK-ERK and estradiol can enhance cyclin D-dependent CDK4/6 activity (Figure 2) 4. In breast cancer, cyclin D1 amplification and CDK4 copy gain are common in luminal and HER2-enriched subtypes but are rare in basal-like tumors with *Rb* loss or mutation and cyclin E1 amplification 5. Numerous preclinical studies have shown that cyclin D1-CDK4/6 is a necessary factor in sustaining the tumorigenic potential of breast cancer cells 6-8.

Given the critical role of CDKs in the cell cycle, it is not surprising that work has been done on developing selective CDKs inhibitors. Pan-CDK inhibitors were the first generation of this class of drugs and were quickly abandoned due to their toxicity profile for normal cells, which led to severe side effects and difficulties to determine an effective dose 9-11. These issues have been overcome by more selective targeting of CDK 4 and 6, which mediate transition from G0/G1 phase to S phase of the cell cycle 12. Selective CDK4/6 inhibitors have been developed and have altered the systemic treatment pattern in breast cancer patients. Preclinically, cell lines that represented luminal estrogen receptor positive (ER-positive) subtype, including those that were human epidermal growth factor receptor 2 (HER2) amplified, were the most sensitive to growth inhibition by palbociclib. Furthermore, strong synergistic effects have been observed when CDK4/6 inhibitors were added to standard anti-estrogen therapies 13. Several randomized clinical trials have shown the application of CDK4/6 inhibitors as a valuable clinical approach (Table 1). As a result, three CDK4/6 inhibitors, including palbociclib (Ibrance), ribociclib (Kisqali) and abemaciclib (Verzenio), have been approved by the US Food and Drug Administration (FDA) for treatment of hormone receptor (HR)-positive/human epidermal growth factor receptor-2 (HER2)-negative advanced or metastatic breast cancer. There is no approval for HER2-positive disease yet 14.

Notably, despite the clinical benefits of CDK4/6 inhibitors, tumor resistance is developing in the metastatic patients and the mechanisms for this resistance are not clear. Preclinical studies are exploring mechanisms of resistance and few clinical studies have reported resistance mechanisms in breast cancer patients. Moreover, studies are currently ongoing aimed to identify combined treatments that might prevent early adaptation of breast cancer cells to the antitumoral effects of CDK4/6 inhibitors. In this review, we focus on the mechanisms of action and efficacy of CDK 4/6 inhibitors and discuss therapeutic approaches to overcome drug resistance.

## 2. The role of CDK4/6 inhibitors in breast cancer

In most adult tissues, differentiated cells are almost always maintained in a G0 phase, these cells are thought to be dormant and wait to enter the cell cycle 4, 15. Appropriate mitogenic stimuli, such as growth factors and hormones, can trigger the cell cycle and induce the progression from G0/G1 phases to S phase 15.

Cell cycle transitions are governed by CDKs' activity 16. In metazoans, the majority of cell cycle entry is controlled by CDK4/6 proteins, which respond to numerous growth regulatory signals 17. CDK4 and CDK6 are serine/threonine kinases that contain a 300-aminoacid catalytic domain usually inactive. When the cells are ready to initiate DNA synthesis in mammalian cells, CDK4/6 complex binds to D-type cyclins (cyclin D1, cyclin D2 and cyclin D3) and mediates progression through the G1 phase (Figure 1) 18. The kinase activity of CDK4/6 is strictly regulated by a plethora of CDK inhibitors (CDKi), which acts to inhibit cell cycle progression under adverse conditions. CKIs are subdivided into two categories, according to its structure and CDK specificity. Members of the INK4 family [p16INK4a (*Cdkn2a*), p15INK4b (*Cdkn2b*), p18INK4c (*Cdkn2c*) and p19INK4d (*Cdkn2d*)] primarily target CDK4 and CDK6 19. The INK4 proteins weaken the binding of D-type cyclins to CDK4/6 and interact with the catalytic domains of CDK4/6 to potently suppress kinase activity 17. In contrast, the Cip/Kip family members [p21Cip1 (*Cdkn1a*), p27Kip1 (C*dkn1b*) and p57Kip2 (*Cdkn1c*)] more extensively interfere with the activities of cyclin D-, E-, A- and B-dependent kinase complexes 19.

When cells pass G1 phase, cyclin D-CDK4/6 is the first complex to become active in G1, which results in the phosphorylation of their downstream target, the Retinoblastoma-associated protein (pRb) (Figure 1) 20. *Rb* is a tumor suppressor that regulates multiple critical cellular activities, including late G1 restriction point, DNA damage response checkpoints, cell cycle exit and differentiation 21. The retinoblastoma family includes three members, Rb/p105, p107 and Rb2/p130, collectively referred to as “pocket proteins” 22. Rb inhibits the expression of many genes normally activated by the E2F transcription factor, a family of protein that regulates genes involved in cell cycle control, mitotic progression and dNTP biosynthesis 20, 22, 23. The hyperphosphorylation of Rb reduces the affinity for E2F, thereby making it possible to activate and transcribe the E2F-target genes required for cell division. Moreover, Rb is also phosphorylated by cyclin E-CDK2 in the late G1 phase 24. Cyclin A2-CDK2 complex phosphorylates proteins such as pocket proteins (Rb, p107, p130) and proteins involved in DNA synthesis, thus promoting the S phase process 25. Cyclin D-CDK4/6 complex also phosphorylates the transcription factor FOXM1 (Forkhead Box M1), which leads to FOXM1-dependent expression of genes that support cellular proliferation and suppress senescence induction 26.

The cyclin D-CDK4/6-INK4-Rb pathway is commonly dysregulated in a wide variety of human cancers, such as sarcoma, glioma, breast tumors, lymphoma, leukaemias and melanoma 27, 28. Many studies have indicated that this pathway plays a vital role in the occurrence, development, precision medicine and prognosis of breast cancer 26, 29. Defects in the principal late-G1 cell-cycle checkpoint regulated by pRb, which may be universal in human cancers, include loss of RB1 protein and deregulation of the CDKs, also through direct over-activation of CDKs or genetic deletion of their inhibitors 30. Cyclin D1 is overexpressed in over 50% of breast cancers 31. Amplification and overexpression of cyclin D1 may contribute to its oncogenicity, and the oncogenic predisposition occurs within luminal tumors, more specifically within Luminal B breast cancers 32-34. Cyclin D1 is a product of the *CCND1* gene, which is a recognized human oncogene 35. *CCND1* amplification and overexpression are involved in breast cancer, lung cancer, melanoma and oral squamous cell carcinoma. *CCND2* or *CCND3* amplification is rare compared to *CCND1* amplification 36, 37. In breast cancer cells, cyclin D expression is enhanced by ligands or mutant activated estrogen receptors, which bind directly to the *CCND1* promoter 26. Estrogen can modulate mitosis by using cyclin D1 as one of its target genes 35. In addition, the canonical RAS-RAF-MEK-ERK pathway and heightened activity of the HER2-PI3K-AKT axis also play a significant role in regulating cyclin D1 gene expression 38, 39. In theory, overexpression of cyclin D1 can induce the hyperactivation of CDK4 and CDK6 40. The expression of CDK4 is essential for the development of breast cancer, while the level of CDK6 is decreased in many breast tumors and in most breast tumor-derived cell lines 41, 42. Yu Q et al. demonstrated that re-expression of human wild-type CDK4 endowed the cells with the ability to form tumors 7.

The effects of CDK4/6 inhibitors are dependent on the presence of a functional RB protein. CDK4/6 inhibitors bind to the ATP-binding pocket existed in protein kinases, and thereby block downstream CDK4/6-mediated phosphorylation of Rb 17. Unphosphorylated Rb still binds to E2F in an inactive complex, which results in the loss of genes that favor cell cycle progression. In this way, cells are arrested at the G1-S checkpoint and impossible to entry cell division 43. *In vivo* trials, palbociclib caused a sustained suppression of tumor Rb phosphorylation, and exhibited significant antitumor efficacy that arrested Rb-positive tumors exclusively in G1, including Rb-positive breast cancer 44. In addition, synergistic activity between cell cycle and anti-estrogen therapies had been observed in breast cancer cell lines 43. As it was specifically in S phase that antiestrogens repress transcription of several ER target genes, an increase in apoptosis was observed when S phase blocked cells were treated with endocrine therapy compared with non-arrested cells. And the use of endocrine therapy in the S phase led to a decrease in cell survival, which was associated with a significant reduction in cyclin D1 transcription 45, 46. Published preclinical studies also supported the synergistic activity between palbociclib and endocrine therapy. Furthermore, palbociclib could reverse acquired resistance to anti-hormone therapy 13.

## 3. Current status of CDK4/6 inhibitors

Over the past few decades, we have witnessed tremendous progress in developing new and effective therapies, particularly through diverting tumor cells from a proliferation phenotype towards a non-division state. According to the important role of CDK4/6 in cell cycle regulation, CDK4/6 inhibitors are the most attractive findings. Prior experience with relatively non-selective pan-CDK inhibitors has led to limited clinical activity and poor safety 47, 48. Highly selective oral CDK4/6 inhibitors palbociclib, ribociclib and abemaciclib can inhibit the proliferation of Rb-positive tumor cells and show dose-dependent growth inhibition in ER-positive breast cancer models 47, 48. All three drugs are small-molecule, ATP-competitive drugs, which bind to the ATP cleft of CDK4 and CDK6. However, abemaciclib buries two fluorine atoms against the back wall of the ATP-binding pocket and appears to bind more readily to the ATP cleft. On the other hand, it forms a hydrogen bond with a catalytic residue (Lys43) that is conserved among kinases, suggesting it binds with less selectivity than ribociclib and palbociclib 49.

Preclinical and clinical studies have shown CDK4/6 inhibitors' efficacy in HR-positive breast cancers. Their cooperative data was the basis for designing clinical trials in ER-positive breast cancers 50. By blocking the aromatase enzyme, the third-generation aromatase inhibitors (AIs, anastrozole, letrozole and exemstane) are initially effective in the treatment of ER-positive tumors. However, *de novo* and acquired resistance remains a barrier to long-lasting clinical responses, particularly in the advanced disease 51, 52. The selective estrogen receptor modulators (SERMs, tamoxifen, toremifene) and selective estrogen receptor downregulators (SERDs, fulvestrant) also face the drug resistance problem 51, 52. One of the key features of CDK4/6 inhibitors is inhibition of cell proliferation in breast cancer cells that have developed resistance to endocrine therapy 52. Combination therapies of CDK4/6 inhibitors with endocrine therapy (exemestane) and everolimus [an inhibitor of mTOR (mammalian target of rapamycin) signaling pathway] have shown significant clinical benefit 53. The clinical trials mentioned in this section are summarized in Table 1. Furthermore, luminal androgen receptor (LAR) subtype of triple negative breast cancer (TNBC) was highly sensitive to CDK4/6 inhibitors, while basal-like TNBC was resistant. Therefore, CDK4/6 inhibitors may be considered as a novel therapeutic approach for TNBC 54.

### 3.1 Palbociclib

Palbociclib is the first CDK4/6 inhibitor to be introduced into clinical practice. It is not only a highly selective inhibitor of CDK4/6, but also equally effective for CDK4 and CDK6. Its peak concentration is between 6 and 12 hours and reaches a stable state within 8 days 55.

The PALOMA-1/TRIO-18 trial was a randomized phase 2 trial designed to evaluate the addition of palbociclib to letrozole therapy in patients who had received no prior treatment for ER-positive, HER2-negative advanced breast cancer (ABC). The combination therapy significantly improved progression-free survival (PFS) compared with single-agent letrozole [20.2 versus 10.2 months, hazard ratio (HR) 0.488, p=0.0004] 53, 56, 57. Based on the results of PALOMA-1 trial, the FDA approved palbociclib (Ibrance) for use in combination with letrozole for the treatment of postmenopausal women with ER-positive, HER2-negative ABC as the first-line therapy for their metastatic disease, on February 3, 2015 56, 58.

Then the PALOMA-2 phase 3 trial confirmed the clinical activity of palbociclib plus letrozole. The median PFS was 24.8 months in the combination therapy, as compared with 14.5 months in the placebo plus letrozole group (HR 0.58, p<0.001) 59. At the same time, 125 patients were enrolled in the QT interval corrected for heart rate (QTc) evaluation substudy by using Fridericia's correction (QTcF), Bazett's correction (QTcB), and a studyspecific correction factor (QTcS). This study demonstrated that when palbociclib administered with letrozole at the recommended therapeutic dosing regimen, QTc prolongation (<480ms) was not a safety concern for palbociclib 60.

Additionally, in the PALOMA-3 randomized phase 3 trial, patients were randomly assigned 2:1 to fulvestrant plus palbociclib or placebo. These patients were hormone receptor (HR)-positive, HER2-negative ABC patients who had relapsed or progressed during previous endocrine therapy in any menopausal status. Median PFS was 9.5 months in the fulvestrant plus palbociclib group and 4.6 months in the fulvestrant plus placebo group (HR 0.46, p<0.0001) 61. On February 19, 2016, the FDA approved palbociclib (Ibrance) for use in combination with fulvestrant for the treatment of women with HR-positive, HER2-negative advanced or metastatic breast cancer (MBC) in the second-line setting. The approval was based on the results of PALOMA-3 trial 61, 62.

Based on these long-term safety analyses of three randomized phase II and III studies (PALOMA1, 2, 3), palbociclib plus endocrine therapy has not shown specific cumulative or delayed toxicities to HR-positive, HER2-negative ABC, supporting the ongoing investigation of palbociclib plus endocrine therapy in early breast cancer (NCT02513394) 63. The PALLAS (NCT02513394) is a randomized phase III trial of palbociclib with standard adjuvant endocrine therapy versus standard adjuvant endocrine therapy alone for HR-positive, HER2-negative early breast cancer. Primary results are expected in 2020 64. Another phase III trials, PENELOPE-B (NCT01864746), is designed to demonstrate that in the background of standard anti-hormonal therapy palbociclib provides superior invasive disease-free survival (iDFS) compared to placebo in premenopausal and postmenopausal women with HR-positive, HER2-normal early breast cancer at high risk of relapse after showing less than pathological complete response to neoadjuvant taxane-containing chemotherapy. Primary results are expected in December 2020 64.

### 3.2 Ribociclib

Ribociclib is another rapidly absorbed inhibitor of CDK4/6, reaching maximal concentration at 3.0-5.0 hours 65. The ribociclib monotherapy dose escalation study (NCT01237236) declared the recommended phase II dose 600 mg/d on 21-of-28-d schedules and the maximum tolerated dose as 900, among 128 patients with Rb+ solid advanced tumors and lymphomas, including 18 breast cancer patients 66.

In MONALEESA-1, the phase II study, postmenopausal women with HR-positive, HER2-negative early breast cancer received letrozole with or without ribociclib. The ribociclib plus letrozole combination was well tolerated and no grade 3/4 adverse events were observed after treatment 67.

MONALEESA-2 was a phase 3 randomized, double-blind, placebo-controlled trial. 668 postmenopausal HR-positive and HER2-negative ABC patients who had not received prior treatment were randomized 1:1 to receive ribociclib plus letrozole or placebo plus letrozole 68. As compared to placebo, the addition of ribociclib improved PFS from 16 months to 25.3 months (HR 0.56, p<0.001) 14, 68. Based on these data, FDA approved ribociclib (Kisquali) in combination with letrozole for the first-line treatment of postmenopausal women with HR-positive, HER2-negative ABC or MBC on March 13, 2017 64, 69.

The MONALEESA-3 phase 3 trial was intended for postmenopausal women with HR-positive, HER2-negative ABC to receive ribociclib or placebo with fulvestrant. The median PFS was 20.5 months in the ribociclib arm versus 12.8 months in the placebo arm (HR, 0.593, p<0.001) 70. On July 18, 2018, ribociclib (Kisquali) was also approved by FDA in combination with fulvestrant for the treatment of postmenopausal women with HR-positive, HER2-negative ABC or MBC, as first-line or second-line therapy on the basis of MONALEESA-3 trial 64.

Recently, the results of the phase III MONALEESA-7 trial were presented. All women with HR-positive, HER2-negative ABC in the study received ovarian function suppression together with oral endocrine therapy (tamoxifen or an aromatase inhibitor) plus ribociclib or not 14. Median PFS was 23.8 months in the ribociclib group versus 13.0 months in the placebo group (HR 0.55, p<0.0001). OS results outcomes were immature, with 89 deaths at the end of the data 71.

### 3.3 Abemaciclib

Abemaciclib is also a highly selective inhibitor of CDK4/6, and may have more complex pharmacological functions, including an effective CDK9 inhibition 49. The breast cancer cell lines treated with LY2835219 showed a concentration-dependent inhibition of pRb, and corresponding arrest of cells in G1 phase, which inhibited proliferation and led to decreased cell number 72.

The first phase II study to report single-agent activity of abemaciclib was MONARCH-1 trial, for heavily treated ER-positive, HER2-negative MBC patients. The primary endpoint of ORR was 19.7%, with an observed clinical benefit rate of 42.4%. The median PFS was 6.0 months 73.

MONARCH 2 was a phase III study of 669 patients with HR-positive, HER2-negative ABC who had progressed during neoadjuvant or adjuvant endocrine therapy. Patients were randomized 2:1 to receive abemaciclib or placebo and fulvestrant. Median PFS was 16.4 months in the abemaciclib plus fulvestrant arm versus 9.3 months in the fulvestrant arm 74. Based on MONARCH 2 trial results, on the September 28, 2017, the FDA approved abemaciclib (Verzenio) in combination with fulvestrant in patients with HR-positive, HER2-negative ABC or MBC as second line therapy. On the same date, in the view of the MONARCH-1 trial results, abemaciclib (Verzenio) also approved by FDA as a monotherapy in patients with HR-positive, HER2-negative ABC or MBC as second or plus therapy in the metastatic setting 64, 75.

MONARCH 3 was a randomized phase III trial. 493 HR-positive, HER2-negative ABC patients who had not received prior treatment, were randomized 2:1 to receive abemaciclib plus anastrozole or letrozole versus placebo plus anastrozole or letrozole. The abemaciclib arm had a significantly longer median PFS than the placebo arm (28.18 versus 14.76 months) 76. On August 17, 2018, FDA approved abemaciclib (Verzenio) in combination with an aromatase inhibitor as the treatment of postmenopausal women with HR-positive, HER2-negative ABC or MBC in the first line, given the results of the MONARCH 3 trial 64, 75.

## 4. Mechanisms of resistance and combined treatment to alleviate drug resistance

CDK4/6 inhibitors are becoming increasingly common in HR-positive, HER2-negative metastatic breast cancer patients and will certainly continue to increase in the future. However, their cytostatic effects are limited by primary and acquired resistance. Currently, there are lots of preclinical data about the mechanisms of *de novo* and acquired resistance to CDK4/6 inhibitors in breast cancer, but little has been demonstrated in clinical settings 77.

### 4.1 Polyclonal RB1 mutations and Loss of Rb function

The effect of CDK4/6 inhibitors on inhibiting tumor cell growth is achieved by blocking the phosphorylation of Rb in the low nanomolar range 13. CDK4/6 inhibitors have been demonstrated to be effective against a variety of human Rb-positive tumors, including breast cancer 77. In human breast cancer cell lines cultured *in vitro* to investigate the effects of palbociclib, higher levels of *RB1* and *CCND1*, and lower levels of *CDKN2A* were found in the sensitive group 13. However, most Rb-negative tumor cells were resistant to CDK4/6 inhibitors 77. Direct analyses of primary tumors reported loss of Rb function in 20% to 35% of breast cancers. Considering the distribution of this direct inactivation, Rb inactivation may be a parameter leading to breast cancer heterogeneity 78.

In breast cancer cell lines, chronic loss of Rb has been associated with the development of a CDK4/6 inhibitor-resistant state 79. The same result was also found in explants derived from human breast tumors 80. To directly explore the functional consequences of *Rb*, knockdown experiments were performed in immortalized mammary epithelia and breast cancer models. The results showed that palbociclib inhibited cell-cycle progression of normal human breast epithelial cells, and its activity mainly occurred through Rb-mediated E2F repression 79. In the following tumor cell lines, such as MDA-MB-231 and more significant levels in MCF-7 cells, Rb deficiency produced a very significant growth advantage in the presence of palbociclib, which had been observed to increase levels of E2F-target genes cyclins A and E 79. These analyses indicated that the cells depended on alternative compensatory signaling pathways for their survival, which function independently of CDK4/6 activity, leading to treatment resistance 79.

Studies had shown that acquired mutation in *RB1* induced resistance to CDK4/6 inhibitor in PDX (from patients with ER-positive breast cancer). After 40 days of ribociclib treatment, tumors began to reproduce under drug stress. Compared to PDX244, which was sensitive to CDK4/6 inhibitors, western blot analysis showed that 4 of 7 CDK4/6-acquired resistant tumors had decreased levels of pRb protein and the E2F target cyclin E2 was continuously expressed. The genomic characteristics of PDX244LR1 (a serial passage of an LEE011-relapsed tumor) showed that RB1 frameshift mutation (*RB1* p.M695fs*26) was obtained. In fact, loss of *Rb* expression was also detected in palbociclib-resistant cell lines *in vitro* 81. In another study, authors derived a new signature of Rb loss-of-function (RBsig) to test whether this might identify palbociclib resistant and sensitive breast cancer cells. They found that the RBsig confirmed there was a poor prognosis for tumors with impaired *Rb* function. And the RBsig helped in discriminating between palbociclib resistant versus sensitive breast cancer cell lines 82.

### 4.2 Hyperactivity of cyclin A/CDK2 or cyclin E/CDK2

CDK4/6-mediated Rb phosphorylation was first detected in mid-G1 phase after induction of cyclin D in mammalian cells entering the division cycle from G0, but prior to activation of cyclin E- and A- dependent CDK2 4. ER-positive breast cancer cell lines were inhibited by palbociclib in culture, but they adapted very quickly as they allowed p27 degradation and subsequent increased in CDK2 activity, which would compensate for the loss of CDK4 activity and led to Rb phosphorylation and proliferation recovery 83. Notably, all populations emerging from extended CDK4/6 inhibition possess increased CDK2 protein and/or loss of p21/p27. As an assembly factor, p27Kip1 is required both for the stabilization and the subsequent activation of cyclin D-CDK4 complex 84. However, p27 must be activated on residue Y88 or Y89 to open or activate the complex. The activated effect of p27 depends on its phosphorylation status. Nonphosphorylated p27 is a stabilized form, which inhibits CDK2 activity as well as CDK4 85.

Thus, ALT (the Brk-SH3 peptide) induction blocks p27 Y88 phosphorylation and then inhibits both CDK4 and CDK2, causing a potent and long-lasting cell-cycle arrest 83, 84. The combination of ALT and PD more potently reduced the activity of CDK2 and CDK4, synergized in cell arrest and increased senescence, and prevented cell recovery when the drug was removed 83. And screening data showed that bone morphogenetic protein (BMP) 4 could inhibit cell growth and synergize with endocrine therapy and CDK4/6 inhibitors. By upregulation of p21, BMP4 enhanced sensitivity to CDK4/6 inhibitors in estrogen-resistant cells (Figure 2) 86. Furthermore, transcriptomic features of BMP4 signaling predicted an improved biological response to the palbociclib combined with an aromatase inhibitor 86. Fangchinoline is an alkaloid with cytotoxic, anti-inflammatory and antioxidant properties. In MCF-7 and MDA-MB-231 cells, the anti-proliferative activity of Fangchinoline was reflected in the downregulation of cyclin D1/D3/E and CDK2/4/6 (Figure 2) 87. High expression levels of MMP-2, MMP-9 and NF-κβ were associated to metastasis of breast tumors. In MDA-MB-231 cells, Fangchinoline inhibited the activation of AKT to increase the level of Iκβ which inhibited the NF-κβ activity and reduced the levels of MMP-2 and MMP-9, thus to inhibit migration of the cells 88. Given the Fangchinoline-induced cell growth inhibition and G1 cell-cycle arrest, combination therapy with CDK4/6 inhibitors may have progress in the next study.

### 4.3 Upregulation of phosphorylated PDK1

3-phosphoinositide-dependent protein kinase 1 (PDK1) is one of the key targets of PI3K signal downstream and also the key upstream kinase of AKT 89. PDK1 phosphorylation is frequently increased and significantly associated with the breast cancer invasiveness. It is worth noting that moderate to high level of phosphorylation on PDK-1 (S241) is retained in high grades and metastatic breast tumors, indicating that phosphorylation and subsequent activation on PDK1 may contribute to aggressive metastasis of breast cancer 89.

Studies confirmed that, in ribociclib-resistant cell lines, the PI3K/PDK1 pathway mediated cell survival and proliferation by up-regulating of AKT and non-AKT targets of PDK1, all of which reached the peak in abnormal cell-cycle progression with emphasis on the presence of CDK4/6 90. Moreover, through increased CDK2/cyclin E/cyclin A, PDK1 promoted cell-cycle progression in CDK4/6-resistant cell lines 90. Another study found that after chronic exposure to palbociclib, E2F-induced G1-S phase regulators such as cyclin E2 or CDK2 persisted, which failed to fully inhibit Rb phosphorylation, resulting in a slight increase in AKT phosphorylation 81. CDK2/cyclin A2 acted as a major physiological kinase and had a role in controlling Akt phosphorylation and carcinogenesis. Notably, CDK2/cyclin A directly phosphorylated AKT1 on its carboxy (C)-terminal region *in vitro* 91. Therefore, it can be demonstrated that early adaptation after exposure to CDK4/6 inhibition can be achieved by PI3K signaling through maintaining the expression of G1-S phase cyclin 81.

PI3K/mTOR inhibitors induced synergistic anti-proliferative and pro-apoptotic effects by inhibiting both CDK4/6/Rb/myc and PI3K/mTOR signaling (Figure 2) 92. Another study showed that cancer cells apoptosis after combined CDK4/6 and PI3K inhibition *in vitro* and patient-derived tumor xenograft (PDX) models. In addition, endocrine therapy, a triple combination of CDK4/6 and PI3K inhibition was more effective in triggering rapid tumor regression in the PDX model 81. Michaloglou C et al. found that when ER-positive breast cancer became resistant to CDK4/6 inhibitors, it still relied on E2F transcription to drive proliferation, which also confirmed the dependence of ER-positive breast cancer cells at this checkpoint 93. At the same time, they demonstrated that inhibition of mTORC1/2 did lead to a decrease in cyclin D1 protein, Rb phosphorylation and E2F-mediated transcription, but did not directly affect ER function. In breast cancer cell lines and xenografts, the combination of mTORC1/2 inhibitors and CDK4/6 inhibitors had a deeper impact on E2F-dependent transcription, which was manifested in more persistent growth arrest and delayed drug-resistant episodes 93.

The RAS-RAF-MEK-ERK pathway is also an important pathway mediating the biological response of the epidermal growth factor receptor (EGFR), which regulates the growth and survival of breast cancer cells. Continuous ERK activation is a necessary condition for progression of G1 into S phase. And the ERK pathway induces cyclin D1 expression through its activation of the AP-1 complex 94, 95. In *KRAS* mutant/*PIK3CA* wild-type cell lines (SW620 and H747), effective downregulation of cyclin D1 expression and cell arrest in G1 phase were detected under the presence of MEK inhibitors 96. Based on these preclinical studies, several combination studies are now broadly interrogating the efficacy and safety of MEK and CDK4/6 inhibitors (Figure 2).

MicroRNAs (miRNAs) are small noncoding RNAs that regulate the translation of mRNA into proteins, and have been thought to be associated with specific molecular subtypes and clinicopathological characters in breast cancer, including miR-126 97. Significant downregulation of miR-126 was evident in breast cancer cell lines. Upregulated expression of miR-126 inhibited cell cycle transforming from G1/G0 to S phase and inhibited insulin receptor substrate-1 (IRS-1) 98. IRS-1, as an adaptor of IGF1R (insulin-like growth factor-1 receptor, which overexpressed in about 70% of breast cancer), played an important role in cell growth and proliferation mainly through activation of the downstream pathways such as PI3K-AKT and RAS-RAF-MAPK pathways 98, 99. After identifying 14 miRNA/drug combinations, miR-126 was the only miRNA that had significant enhanced effects in combination with CDK4/6 or PIK3CA inhibitors *in vitro* 100.

### 4.4 Acquired CDK6 amplification

After prolonged exposure to the CDK4/6 inhibitor abemaciclib, clones harboring CDK6 amplification emerged, resulting in a reduced response of breast cancer cells to the growth-inhibitory effects of CDK4/6 inhibitors 101. Unexpectedly, overexpression of CDK4 had never been observed in these models, and further experiments showed that enforced overexpression of CDK4 did not promote drug resistance 101. Yang C et al. speculated that inhibitor response was influenced by the partner cyclin or other components of the complex. CDK6 bound to cyclin D3 preferentially and the complex was more resistant than the cyclin D1-CDK4 complex. Above reasons raised the possibility that more powerful inhibitors for CDK6 might have greater clinical interest for acquired resistance patients 101.

## 5. Methods to explore the mechanism of drug resistance

Liquid biopsy is one of the revolutionary technologies involved in the detection and isolation of circulating tumor cells, circulating tumor DNA and exosomes, which extracted from plasma or other body fluids can serve as a source of genomic and proteomic information for cancer patients 102. Circulating cell-free DNA has potential innovative applications in the diagnosis and management of cancer patients. Circulating blood contains millions of copies of the genome which is divided into short fragments, a small fraction of which is circulating tumor DNA (ctDNA) in cancer patients 103. A study had identified first detectable multiple de novo somatic *RB1* mutations in circulating tumor DNA (ctDNA) after 5, 8 and 13 months of exposure to CDK4/6 inhibitors (palbociclib, ribociclib), respectively, in three MBC patients. Their appearance dynamics suggested the mutations were related to the acquisition of resistant phenotype 104. In a published case report, a patient with ER-positive breast cancer was treated with letrozole, everolimus, and palbociclib. After 11 months of treatment, *RB1* mutation was caught in available ctDNA tests, which suggested an acquired resistance to palbociclib 105. In another clinical study, HR-positive, HER2-negative patients received ctDNA evaluation at 3 months after chemotherapy treatment (except one who received chemotherapy plus letrozole). Patients (who had PFS < 3 months) exhibited increased mutation frequencies in *TERT*, *FAT1*, *RARA*, and *ERBB4*, while patients (who had progression with PFS > 3 months) had increased mutations in *PIK3CA*, *TP53*, *NOTCH2*, and *MLL3*. This suggested a distinct mechanism for drug resistance between HR-positive patients with different disease progression time 106.

Tim Forshew et al. applied tagged-amplicon deep sequencing (TAm-Seq) to detect abundant and rare mutations in circulating DNA in plasma of breast cancer patients. This sequencing method allowed it to monitor changes in tumor burden by sampling only patient plasma over time 103. Quantification of allele fractions in plasma identified increased representation of mutant alleles associated with emergence of therapy resistance. In breast cancer patients, research results included an activating mutation in *PIK3CA* following treatment with paclitaxel, truncation mutation in *MED1* (an ER co-activator involved in tamoxifen resistance) following treatment with tamoxifen and trastuzumab, and increased splicing mutation in *GAS6* following treatment with lapatinib and capecitabine. These data demonstrated the possibility that exome-wide analysis of ctDNA to identify mutations associated with acquired drug resistance in breast cancer 107. For another technique, cfDNA-targeted NGS had the potential to monitor targeted therapeutic responses through mutations and gene amplification, which could be used to monitor response and clonal dynamics during treatment in MBC 108. In the next clinical studies, liquid biopsy of breast cancer patients treated with CDK4/6 inhibitors may be a crucial method for detecting the mechanisms of drug resistance.

## 6. Combined treatment with anti-HER2 therapy or immunotherapy

Studies have shown that HER2 dimerizes with other HER2-family partners and activate intracellular proliferative pathways, causing an aggressive clinical behavior 109. Therefore, anti-HER2 therapy has led to dramatic improvements in survival in both early and advanced HER2-positive settings. Yet nearly all patients eventually progress on anti-HER2 therapy due to drug resistance 110. In ER-positive, HER2-positive cancer cells, cyclin D1/CDK4 mediated resistance to anti-HER2 therapy, and CDK4/6 inhibitors were active both as single agents and in combination with trastuzumab *in vitro* studies (Figure 2) 111, 112. Due to these preclinical studies and the great success in advanced ER-positive disease, studies combining CDK4/6 inhibitors and anti-HER2 therapy in “triple positive” patients are rapidly evolving 113. Now there are already many trials investigating this combination both in the neoadjuvant and metastatic settings. In the PATINA trial (NCT02947685), ER-positive, HER2-positive patients will receive first-line induction chemotherapy with trastuzumab/pertuzumab, followed by a maintenance therapy: endocrine therapy/trastuzumab/pertuzumab with or without palbociclib. The PATRICIA trial (NCT02448420) will include trastuzumab-resistant patients after 2-4 lines of anti-HER2 therapy, using different cohorts: ER-negative patients receive trastuzumab/palbociclib and ER-positive patients are randomized to receive trastuzumab/palbociclib with or without letrozole. Another advanced setting, monarcHER trial (NCT02675231) includes ER-positive, HER2-positive patients with at least 2 lines of previous therapy, who are randomized to receive trastuzumab/chemotherapy, trastuzumab/abemaciclib or trastuzumab/abemaciclib/fulvestrant 114.

**Figure 3:**
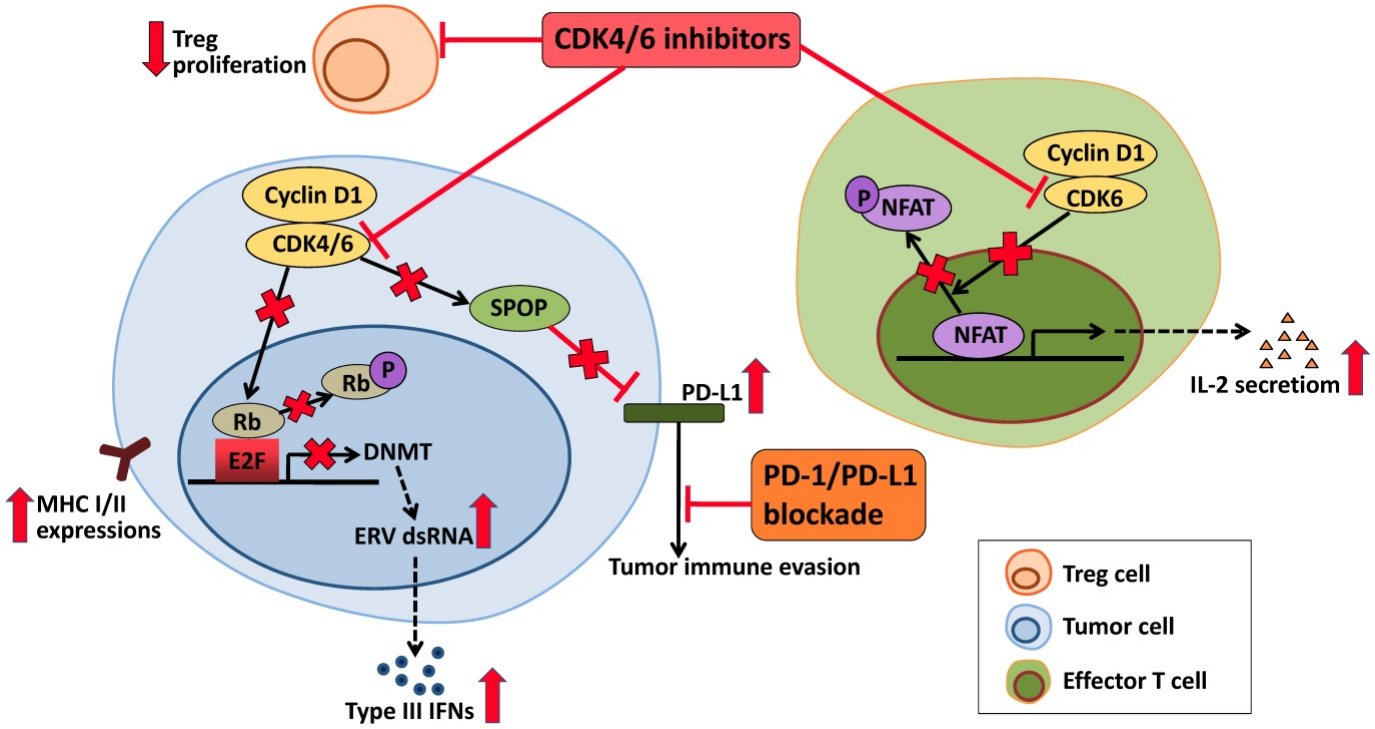
** Effects of CDK4/6 inhibitors in anti-tumor immunity.** In tumor cells, CDK4/6 inhibitor upregulates MHC I/II at tumor cell surface via reduced activity of the DNMT and induction of Type III IFNs, which may activate the anti-tumor activity of immune cells. In response to CDK4/6 inhibitor, the effector T cells increase the activity of NFAT and produce cytokines that can also enhance the anti-tumor immunity. Proliferation of Treg cells is suppressed by CDK4/6 inhibitor. While CDK4/6 inhibitor also upregulates the level of PD-L1. Therefore, CDK4/6 inhibitor may be combined synergically with PD-1/PD-L1 blockade in the clinic.

Interestingly, studies had shown that CDK4/6 inhibitors not only induced tumor cell cycle arrest, but also played a role in regulating mitogenic kinase signaling, inducing senescence and promoting anti-tumor immunity 115, 116. Thus, the prospects for CDK4/6 inhibitor-immunotherapy combinations are also promising. CDK4/6 inhibitors enhance antigen presentation, which results from suppression of Rb-E2F axis followed by downregulation of DNA methyltransferase DNMT, induction of endogenous retroviral genes (ERVs), increased levels of double-stranded RNA (dsRNA) and type III interferon (IFNs) molecules 117. Proliferation of immunesuppressive regulatory T cells (Tregs) is suppressed by CDK4/6 inhibitors in the tumor microenvironment 118. In addition, CDK4/6 inhibitors can also enhance the anti-tumor immune response by upregulating the activity of NFAT and the level of cytokines (IL-2) in the effector T cells 118. Cyclin D-CDK4 complex increases SPOP abundance, leading to reduction in the level of PD-L1. Therefore, in tumor cells, CDK4/6 inhibitors lower SPOP and promote expression of PD-L1, causing tumor immune evasion 119, 120. All of these activities of CDK4/6 inhibitors synergize with PD-L1 blockade to further enhance immune activation (Figure 3). Study has demonstrated that although anti-PD-L1 monotherapy had very modest effects, CDK4/6 inhibitors showed combinatorial benefit when combined with anti-PD-L1 therapy 117. A complete overview of the ongoing clinical trials is given in Table 2.

## 7. Conclusions

Because the CDK4/6 plays an important role in the development and progression of breast cancer, CDK4/6 inhibitors have revolutionized the treatment of metastatic breast cancer. In combination with endocrine therapies, CDK4/6 inhibitors have become a new standard of care for patients with ER-positive breast cancer. Current research on the resistance mechanisms of CDK4/6 inhibitors are only at the beginning stage. With the extensive application of CDK4/6 inhibitors in clinical practice, the resistance mechanisms will become a hot spot. More precise researches are needed to guide individualized treatment and combination with other drugs.

## Figures and Tables

**Table 1:** Reported clinical trials investigating CDK4/6 inhibitors in breast cancer

Trial name	Treatment arms	Setting	Primary endpoint	PFS	ORR (measurable disease)	CBR (intention-to-treat population)	G3/G4 adverse events (≥2%)
PALOMA-1/TRIO-18 56, 57	1.Palbociclib + letrozole 2. Letrozole	1st line	PFS	1. 20.2 months 2. 10.2 months (HR 0.488; 95% CI 0.319-0.748; p=0.0004)	1. 55% (95% CI 43-68) 2. 39% (95% CI 28-52)	1. 81% (95% CI 71-89) 2. 58% (95% CI 47-69)	54% neutropenia, 19% leukopenia, 6% anaemia, 4% fatigue, 4% diarrhoea, 2% nausea, 2% thrombocytopenia, 2% nausea, 2% dyspnoea, 2% bone pain
PALOMA-2 59	1.Palbociclib + letrozole 2.Placebo + letrozole	1st line	PFS	1. 24.8 months 2. 14.5 months (HR 0.58; 95% CI 0.46-0.72; p<0.001)	1. 55% (95% CI 49.9-60.7) 2. 44% (95% CI 36.9-52.2)	1. 85% (95% CI 81.2-88.1) 2. 70% (95% CI 63.8-76.2)	66% neutropenia, 25% leukopenia, 5% anaemia, 2% febrile neutropenia, 2% fatigue, 2% asthenia, 2% thrombocytopenia
PALOMA-3 61	1.Palbociclib + fulvestrant 2.Placebo + fulvestrant	2nd line	PFS	1. 9.5 months 2. 4.6 months (HR 0.46; 95% CI 0.36-0.59; p<0.0001)	1. 25% (95% CI 19.6-30.2) 2. 11% (95% CI 6.2-17.3)	1. 67% (95% CI 61.3-71.5) 2. 40% (95% CI 32.3-47.3)	65% neutropenia, 28% leukopenia, 3% anaemia, 3% thrombocytopenia, 3% increased AST, 2% increased ALT, 2% fatigue, 2% infections, 2% hypertension
MONALEESA-2 14, 68	1. Ribociclib + letrozole 2. Placebo + letrozole	1st line	PFS	1. 25.3 months 2. 16.0 months (HR 0.56; 95% CI 0.43-0.72; p<0.001)	1. 53% (95% CI 46.6-58.9) 2. 37% (95% CI 31.1-43.2)	1. 80% (95% CI 75.3-84.0) 2. 73% (95% CI 68.0-77.5)	59% neutropenia, 21% leukopenia, 9% increased ALT, 6% increased AST, 4% infections, 4% vomiting, 2% fatigue, 2% nausea, 2% back pain
MONALEESA-3 70	1. Ribociclib + fulvestrant 2. Placebo + fulvestrant	1st and 2nd line	PFS	1. 20.5 months 2.12.8 months (HR 0.593; 95% CI 0.48-0.73; P<0.001)	1. 41% (95% CI 35.9-45.8) 2. 29% (95% CI 22.1-35.3)	1. 70% (95% CI 66.2-74.3) 2. 63% (95% CI 56.7-68.9)	53% neutropenia, 14% leukopenia, 6.6% increased ALT
MONALEESA-7 71	1. Ribociclib + tamoxifen or NSAI + goserelin 2. Placebo + tamoxifen or NSAI + goserelin	1st line	PFS	1. 23.8 months 2. 13.0 months (HR 0.55; 95% CI 0.44-0.69; p<0.0001)	1. 51% (95% CI 45-57) 2. 36% (95% CI 31-42)	1. 79% (95% CI 75-84) 2. 70% (95% CI 65-75)	61% neutropenia, 14% leukopenia, 5% increased ALT, 4% increased AST 3% anaemia, 3% hypertension
MONARCH-1 73	Abemaciclib	2nd line and plus	ORR	6.0 months (95% CI 4.2-7.5)	19.7% (95% CI 13.3-27.5)	42.4% (95% CI 33.9-51.3)	28% leucopenia, 27% neutropenia,20% diarrhea, 13% fatigue, 5% nausea, 5% hypokalemia, 4% increased ALT, 3% decreased appetite, 3% hyponatremia, 2% abdominal pain, 2% thrombocytopenia
MONARCH- 2 74	1. Abemaciclib + fulvestrant 2. Placebo + fulvestrant	2nd line	PFS	1. 16.4 months 2. 9.3 months (HR 0.553; 95% CI 0.449-0.681; p<0.001)	1. 48% (95% CI 42.6-53.6) 2. 21% (95% CI 15.1-27.6)	1. 72% (95% CI 68.0-76.4) 2. 56% (95% CI 49.5-62.6)	27% neutropenia, 13% diarrhoea, 9% leukopenia, 7% anaemia, 4% increased ALT, 3% fatigue, 3% nausea, 3% thrombocytopenia, 3% dyspnoea, 3% abdominal pain, 2% increased AST
MONARCH- 3 76	1. Abemaciclib + NSAI 2. Placebo + NSAI	1st line	PFS	1. 28.18 months 2. 14.76 months (HR 0.540; 95% CI 0.418-0.698; p=0.000002)	1. 61% (95% CI 55.2-66.9) 2. 46% (95% CI 37.0-53.9)	1. 78% (95% CI 73.6-82.5) 2. 72% (95% CI 64.6-78.4)	24% neutropenia, 10% diarrhea, 9% leucopenia, 7% anemia, 6% increased ALT, 4% increased AST, 2% blood creatinine increased

Abbreviations: PFS: progression-free survival; ORR: objective response rate; CBR: clinical benefit rate; HR: hazard ratio; CI: confidence interval; NSAI: non-steroidal aromatase inhibitors; AST: aspartate aminotransferase; ALT: increased alanine aminotransferas.

**Table 2:** Ongoing clinical trials in combination with anti-HER2 therapy or immunotherapy. ClinicalTrials.gov April 2019.

Clinical trials.gov identifier	Phase	Recruitment status	Therapy	Breast tumor type	Estimated enrollment	Primary endpoint
**Palbociclib**						
NCT01976169	ⅠB	Recruiting	PD-0332991 + T-DM1	HER2+ ABC	17	- MTD - DLT
NCT03054363	ⅠB/Ⅱ	Active, not recruiting	Tucatinib + palbociclib + letrozole	HR+/HER2+ locally advanced unresectable or metastatic breast cancer	25	- Phase Ⅰ: AE - Phase Ⅱ: PFS
NCT03709082	Ⅰ/Ⅱ	Recruiting	Palbociclib + letrozole + T-DM1	Trastuzumab refractory ER+/HER2+ MBC	62	ORR
NCT03304080	Ⅰ/Ⅱ	Recruiting	Anastrozole + palbociclib + trastuzumab + pertuzumab	HR+/ HER2+ MBC	36	- DLT - MTD - CBR
NCT02907918 PALTAN	Ⅱ	Recruiting	Palbociclib + letrozole + trastuzumab	Stage Ⅱ-Ⅲ ER+/HER2+ BC	48	pCR rate
NCT02448420 PATRICIA	Ⅱ	Recruiting	1. Palbociclib + trastuzumab 2. Palbociclib + trastuzumab.+ letrozole	Postmenopausal previously-treated locally HER2+ ABC or MBC	138	PFS
NCT02530424 NA-PHER2	Ⅱ	Active, not recruiting	1.Trastuzumab + pertuzumab + palbociclib +fulvestrant 2.Trastuzumab + pertuzumab + palbociclib	Invasive unilateral non metastatic ER+/ HER2+ BC	102	- Serial measures of Ki67, - Serial measures of apoptosis
NCT02774681	Ⅱ	Active, not recruiting	Palbociclib + trastuzumab	HER2+ MBC with brain metastasis	12	Radiographic response rate in the CNS
NCT03147287 PACE	Ⅱ	Recruiting	1. Fulvestrant 2. Fulvestrant + palbociclib 3. Fulvestrant + palbociclib + avelumab	HR+/HER2- MBC that has previously stopped responding to prior palbociclib and endocrine therapy.	220	PFS
NCT02947685 PATINA	Ⅲ	Recruiting	1. Palbociclib + trastuzumab/pertuzumab + letrozole, anastrozole, exemstane or fulvestratnt 2.Trastuzumab/pertuzumab + letrozole, anastrozole, exemstane or fulvestrant	HR+/HER2+ MBC	496	PFS
**Ribociclib**						
NCT02657343	ⅠB/Ⅱ	Recruiting	1. Ribociclib + Trastuzumab 2. Ribociclib + T-DM1 3. Ribociclib + Trastuzumab + fulvestrant	HER2+ ABC or MBC	86	- MTD - CBR
**Abemaciclib**						
NCT02057133	ⅠB	Recruiting	Abemaciclib + trastuzumab + pertuzumab +loperamide dose escalation	MBC	198	Number of participants with AE
NCT02675231 monarcHER	Ⅱ	Active, not recruiting	1. Abemaciclib + trastuzumab + fulvestrant 2. Abemaciclib + trastuzumab 3.Trastuzumab + chemotherapy	HR+/ HER2+ locally ABC or MBC	225	PFS

Abbreviations: ER+: estrogen receptor-positive; HR+: hormone receptor-positive; HER2+: human epidermal growth factor receptor 2-positive; MBC: metastatic breast cancer; ABC: advanced breast cancer; BC: breast cancer; AE: adverse events; PFS: progression-free survival; ORR: overall response rate; MTD: maximum tolerated dose; DLT: dose-limiting toxicity; CBR: clinical benefit rate; pCR: pathologic complete response; CNS: central nervous system.
